# MEA Preparation for Direct Formate/Formic Acid Fuel Cell—Comparison of Palladium Black and Palladium Supported on Activated Carbon Performance on Power Generation in Passive Fuel Cell

**DOI:** 10.3390/membranes10110355

**Published:** 2020-11-19

**Authors:** Adrianna Nogalska, Andreu Bonet Navarro, Ricard Garcia-Valls

**Affiliations:** 1 Eurecat, Centre Tecnològic de Catalunya, C/Marcel·lí Domingo, 43007 Tarragona, Spain; andreu.bonet@eurecat.org (A.B.N.); ricard.garcia@eurecat.org (R.G.-V.); 2 Department of Chemical Engineering, Universitat Rovira I Virgili, Av. Països Catalans, 26, 43007 Tarragona, Spain

**Keywords:** formate fuel cell, formic acid fuel cell, palladium electro-catalyst, MEA, catalyst coated membrane

## Abstract

Membrane electrode assemblies (MEAs) with palladium catalysts were successfully prepared by using a home-made manual pressing system with Nafion glue application that contributed to a decrease of additional energy consumption. The catalyst coated membranes were prepared with supported palladium on activated carbon (PdC) and unsupported palladium black (PdB) for comparison. The performance of passive, air breathing, functioning under ambient conditions and with low concentration (1 M) formate/formic acid fuel cell was evaluated. Based on polarization curves, the best result was obtained with carbon supported catalyst and HCOOK fuel, achieving 21.01 mW/mg_Pd_. Still, constant current discharge with PdC showed an energy generation efficiency of 14% with HCOOH over 3% with HCOOK caused by lower potassium ion conductivity and its permeability through the proton exchange membrane. The faradic efficiency of conversion in the cell is equal to the overall energy efficiency and makes the cell self-sufficient.

## 1. Introduction

Nowadays, the capture and conversion of CO_2_ has become a main social, economic and technologic priority due to the climate change emergency. For CO_2_ removal to become cost-effective, it is necessary to develop strategies to convert it into usable products. One of the simplest and arguably less energy demanding CO_2_ reduction paths involves its conversion to formic acid. This way, renewable energy, which is intermittent and unpredictable, could be stored as formic acid without any additional CO_2_ released into the atmosphere. Despite being easy to transport and store for long times, the use of formic acid in fuel cells has rarely been investigated as compared to hydrogen and direct alcohol fuel cells, probably due to its energy density (2.1 kWh/L) which is lower than that of methanol (5.9 kWh/L). However, formic acid has numerous advantages: (i) it is a non-flammable liquid at ambient temperature, (ii) allows for easy storage and (iii) the formic acid cross over the proton exchange membrane is six times lower compared to methanol [1], that means that it provides better mass transport improving energy density [2]. The disadvantage of the acid over its salt is its corrosive nature. This can be overcome by use of salt, such as potassium formate, with the same high theoretical cell potential (1.45 V) and fast oxidation kinetics. Thus, the potential use of formate salt in fuel cells has gained a lot of attention in the scientific community [3].

The heart of the fuel cell, specifically the proton exchange membrane fuel cell, is the membrane electrode assembly. It is a combination of the gas diffusion layers and catalysts for redox reactions on the anode and cathode with a proton exchange membrane in a sandwich structure [4]. Selection of the catalyst is crucial and mainly depends on the fuel used in the system.

Gao et al. [5] studied the possible use of a blended fuel of formate and formic acid on platinum. The authors show that the mechanism of the oxidation of mixed fuel is through direct oxidation, while HCOOH alone undergoes oxidation through an indirect mechanism that can lead to poisoning. Still, they reported achieving higher current densities when using formate compared to the acid alone.

Initially, researchers were using platinum catalyst for the formate/formic acid oxidation, as it is used in direct alcohol fuel cells, yet studies show that palladium has better performance, it is cheaper (according to prices of metal catalysts in Sigma Aldrich on October 2020) and it is rarely poisoned by CO, increasing the stability of the catalytic reaction. Bartrom et al. [6] found that formate oxidizes rather efficiently and with no strong bound intermediate on palladium. Catalysts are often supported on activated carbon providing a high contact area, excellent electron conductivity, and improved mass transfer allowing lowering of noble metal usage [7]. Palladium, when supported on activated carbon, can give high reactivity with highly concentrated HCOOH [8].

The main objective of this work is to compare the performance of passive, air breathing formate/formic acid fuel cells using two catalysts, palladium black and palladium supported on activated carbon, with two different fuels, HCOOH and HCOOK, at ambient conditions (25 °C, 1 bar) using low fuel concentration (1 M) to demonstrate which catalyst is most efficient for future commercialization under those conditions. The performance towards formate/formic acid oxidation in a fuel cell system was evaluated by polarization curves followed by constant current discharge. Moreover, X-ray diffraction of the catalysts was performed to describe their crystalline structure, size and to understand the catalysis mechanism. The list of abbreviations can be found in Supplementary Material Table S1.

## 2. Materials and Methods

Parts of membrane electrode assembly (MEA), such as: Toray paper 060, TGP-H-060, as gas diffusion layer (GDL); Nafion membrane 117; and cathode, cloth gas diffusion electrode (GDE) 4 mg/cm^2^ PtB; were purchased from Fuel Cell Store (https://www.fuelcellstore.com/, College Station, Texas, USA). Both catalysts, palladium black (surface area 40–60 m^2^/g, 99.95% trace metals basis) and palladium on carbon (extent of labeling: 30 wt.% loading, matrix activated carbon support), and Nafion glue (Nafion 117 solution 5% in a mixture of lower aliphatic alcohols and water) were purchased from Sigma Aldrich (Madrid, Spain). Isopropanol used in catalytic ink preparation was purchased from Scharlau (Barcelona, Spain). Hydrogen peroxide 30% (*v*/*v*) (Sigma-Aldrich, Madrid, Spain) and 95–97% H_2_SO_4_ (Serviquimia, Tarragona, Spain) were used to prepare cleaning solutions for the Nafion membrane. Potassium formate (reagent plus 99%) and formic acid (for synthesis) used as fuels were purchased from Sigma Aldrich (Madrid, Spain).

**Figure 1:**
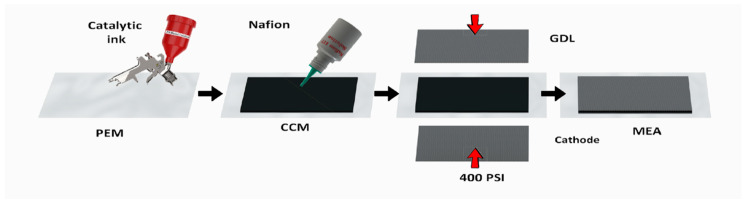
Schematic illustration of membrane electrode assembly (MEA) fabrication.

Figure 1 shows a schematic description of the membrane electrode assembly preparation starting from the catalyst application. The anode was prepared by air brushing (3 bar) of Pd-based catalyst, PdB or PdC, on 9 cm^2^ of a 16 cm^2^ previously cleaned Nafion membrane (Figure S1) to create a catalyst coated membrane (CCM). The catalytic ink was composed of palladium catalyst wetted by Milli-Q water, i-propanol and Nafion glue. The solid part was 6% wt of the total ink while the catalyst to Nafion proportion was 10:1.

Before the deposition, the ink was sonicated (Ultrasons Selecta 3000683 (Selecta, Barcelona, Spain), 50/60 kHz, 110 W) for 30 min. For the MEA preparation, (i) Toray carbon paper (9 cm^2^ GDL), (ii) anode CCM (Nafion membrane 16 cm^2^ containing sprayed catalyst of 9 cm^2^) and iii) commercial cathode (cathode cloth GDE 4 mg/cm^2^ PtB) were glued with iv) 100 µL of Nafion 117 solution 5% and pressed for 2 h with 400 psi. The pressure was applied by a manual home-made cell (Figure S2) and it was measured with the load compression cell distributed by SENSING, S.L (AEP transducers, Modena, Italy).

**Figure 2:**
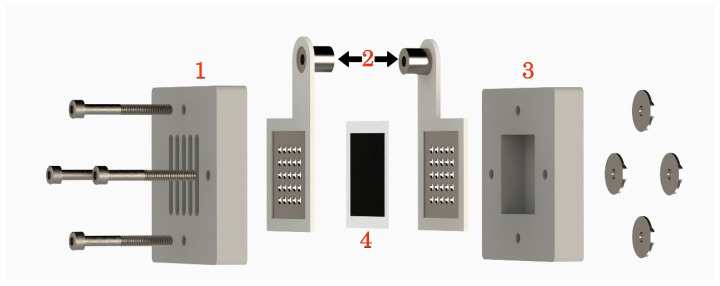
Fuel cell exploded view. 1. cathodic compartment (PMMA), air entrance; 2. current collectors, stainless steel covered with silicon gasket; 3. anodic compartment, fuel reservoir; 4. MEA. (PMMA).

In all measurements, either 1.0 M formic acid or 1.0 M potassium formate were used as fuel source on the anode side with capacity of 7.2 mL in the passive fuel cell (Figure 2). Ambient air was used as oxygen source for the cathodic compartment. Fuel cell Monitor 3.0 (https://www.fuelcellstore.com) was used for the electrochemical characterization of fuel cells. Polarization and corresponding power generation curves were obtained through automatic mode starting from open circuit potential (OCP), whereas the constant-current discharge was measured in manual mode. The working current in galvanostatic conditions was chosen at maximum power point based on the characteristic polarization curves of each system. Additionally, we performed measurements on 20 mA in all systems (3 mA/mg for PdB; 10 mA/mg for PdC). The galvanostatic tests were stopped when cell voltage reached 0.00 V. All measurements were performed at 25 °C and 1013 hPa. The cells were cleaned with Milli-Q water and 1% H_2_SO_4_ between experiments. Current and power density were normalized by weight of palladium for better interpretation of results.

The power production efficiency was calculated based on the theoretical faradic efficiency of conversion and experimental results from galvanostatic measurements by integration of the power vs. time function. Theoretically, the maximum energy our cells could produce was 0.5092 Wh with 100% efficiency. As the fuel cell (FC) system is passive, air breathing and functioning under ambient conditions, the faradic efficiency of conversion is equal to the overall energy efficiency [9].

The characterization of palladium catalysts’ crystalline structures was done by X-ray diffraction (XRD) (Siemens D5000 diffractometer; Bragg-Brentano para focusing geometry and vertical θ-θ goniometer, Aubrey, Texas, USA). The angular 2θ diffraction range was between 5 and 70° Data was collected with an angular step of 0.05° at 3 s/step and sample rotation. Cukα radiation was obtained from a copper X-ray tube operated at 40 kV and 30 mA. Obtained spectra were analyzed with High Score Plus software. Based on the obtained diffractograms, particles size was calculated with TOPAS 6.1 software.

## 3. Results and Discussion

All the parameters of the fuel cell tests are gathered in Table 1. The experiments were performed in passive mode and ambient conditions with no additional energy consumption. Moreover, the use of Nafion glue for the MEA preparation and use of home-made manual pressing system allowed not using heat, contributing to the decrease in the energy consumption.

**Figure 3:**
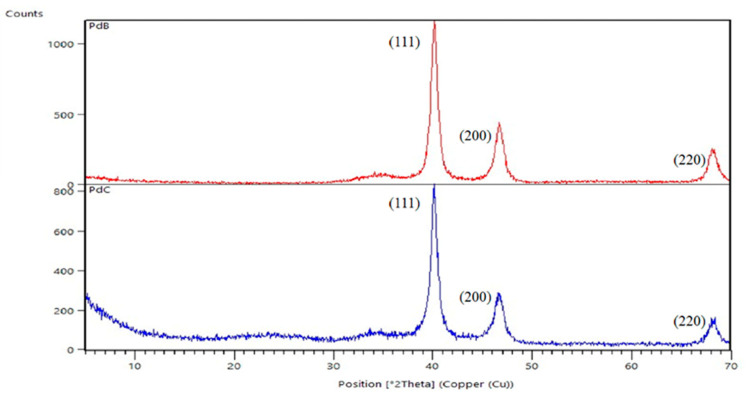
Diffractograms of catalysts.

Palladium black (PdB), was just precipitated palladium, while PdC corresponds to 30% of palladium supported on activated carbon. Figure 3 presents the X-ray diffractograms of the two commercial catalysts. The analysis confirmed the typical face centered cubic structure of crystalline palladium particles [10]. Diffraction peaks at 2Θ = 40.2, 46.6 and 67.9, which represent the Bragg reflections from the (111), (200) and (220) planes, which are present in the diffraction patterns of both diffractograms. PdB gives higher intensity, probably because of its purity. Activated carbon was found to have a highly amorphous state and low crystallinity [11], thus it was not detected with XRD. Furthermore, the size of the catalyst’s particles were calculated to be 7.8 ± 0.2 and 7.1 ± 0.1 nm for PdB and PdC, respectively, thus larger size and the same larger surface area should have a positive impact on the PdB performance.

**Figure 4:**
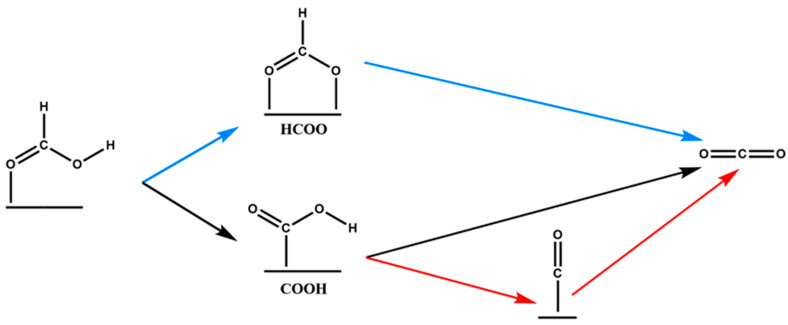
A general formic acid oxidation mechanism on Pd and Pt anodes of direct formic acid fuel cells (DFAFCs) by Elnabawy et al. Paths: blue, direct via formate; black, direct via carboxyl; and red, indirect via carboxyl. Reproduced from [12].

Elnabawy et al. [12] studied the selectivity of platinum and palladium catalysts towards formic acid oxidation to CO_2_. The suggested anodic reaction mechanisms are depicted in Figure 4: direct via formate (blue), direct via carboxyl (black) and indirect via carboxyl (red) through CO*. Pd (100) is mainly catalyzing the reaction through carboxyl and might get slightly poisoned by CO* but it does not lead to permanent deactivation due to its easy removal from the surface. Pd anodes generally offer lower tendency to get poisoned by CO* than Pt anodes. On the other hand, Pd (111) can catalyze the reaction through both paths, formate and carboxyl; however, the formate group is becomes stabilized and, as result, the HCOO* path has a higher contribution to the overall activity on Pd (111).

**Figure 5:**
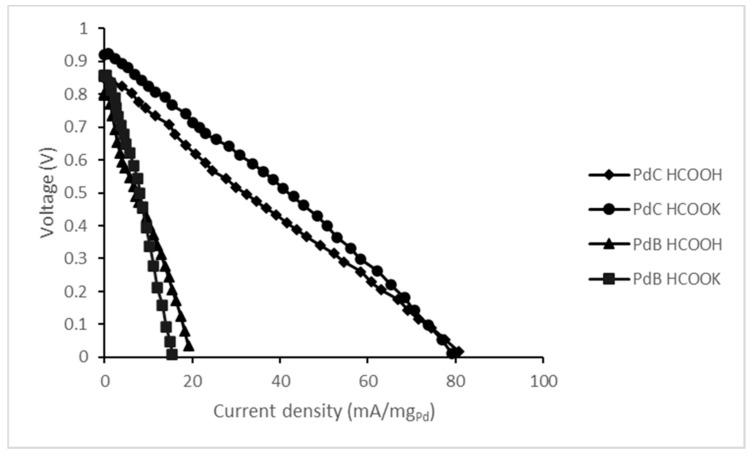
Polarization curves of the developed formate/formic acid fuel cells.

Performance results of the prepared MEAs with different fuels in terms of polarization curves are shown in Figure 5. Based on the polarization curves we can say that PdC performs better in OCP and gives higher current density as well. This might be ascribable to the better gas transport properties of PdC. The use of activated carbon for the Pd support provides a high contact area between catalyst and reagents. Additionally, PdC particles are much smaller than PdB and they contain only 30% of palladium on their surfaces. When we sprayed unsupported palladium, we noticed that it tends to agglomerate (Figure S3), which may cause an increase in the mass transfer resistance of gasses and fuels, which agrees with the literature [13,14].

Table 2 includes the open circuit potential values of all studied cases derived from Figure 3. The electrochemical potential of formic acid (or formate salt) oxidation reaction is as follows:

Anode: HCOOH (aq) → CO_2_ (g) + 2H^+^ (aq)+ 2e^−^    –0.22 V (1)

Cathode: 1/2O_2_ (g) + 2H^+^ (aq)+ 2e^−^ → H_2_O (l)      1.23 V (2)

Net reaction: HCOOH (aq) + 1/2O_2_ (g)→ CO_2_ (g) + H_2_O (L)  1.45 V (3)

Despite the theoretical OCP being 1.45 V, according to the net reaction of formate/formic acid oxidation (3), the experimental results were lower. This difference between the theoretical potential value and the measured value is imputable to the use of air as oxygen source instead of pure oxygen as the catalyst type should not influence the OCP. Moreover, HCOOK has a slightly higher OCP compared to the acid for both studied catalysts. The better performance of the salt might be a result of its higher dissociation. Indeed, the dissociation constant for HCOOK is 0.19 mol/dm^3^, while for HCOOH it is only 1.46 × 10^−4^ mol/dm^3^ [15]. Based on XRD analysis we state that both catalysts used in this study are Pd (111) where formate anions are precursors for the oxidation reaction.

**Figure 6:**
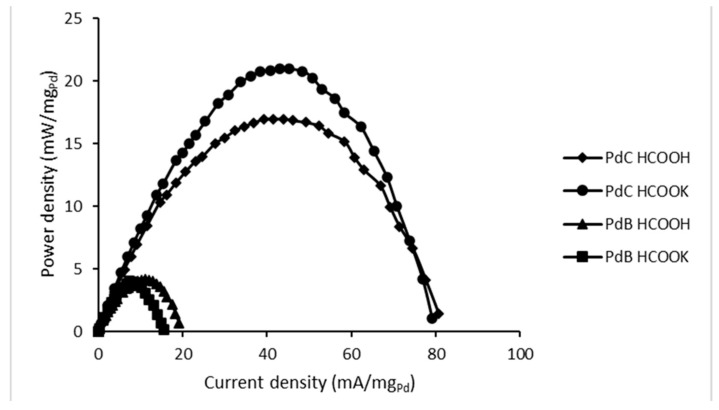
Formate/formic acid fuel cells’ performances.

Figure 6 includes the values of power density as a function of current density for all studied FC configurations. Additionally, in Table 3 all data of maximum power peak is gathered based on the cells’ performances from Figure 6. Once again, the use of PdC results in better performance and higher power density compared to PdB. The maximum power peak with PdC appears at similar current density for both fuels, with the peak being higher for HCOOK. Thus, from a kinetic standpoint, its oxidation reaction is clearly more favorable. PdB reveals the opposite tendency: the maximum power density is almost equal for both studied fuels, but the current density on the peak is higher for HCOOH.

Table 4 represents the comparison of obtained results of power density normalized to catalyst area with literature findings. It summarizes the results of the formate/formic acid fuel cells working in different conditions, such as temperature, oxygen source or fuel concentration and addition of supporting electrolyte in the form of an alkaline solution. In our system, we are evaluating the performance of an FC in very mild and ambient conditions using a fuel of low molarity without an electrolyte, and we could still detect power even with an amount of catalyst as low as 1 mg/cm^2^. Considering that no additional energy was used for solution circulation, pure oxygen pumping or heating it is a promising start for development of self-sufficient fuel cells for CO_2_ recycling.

**Figure 7:**
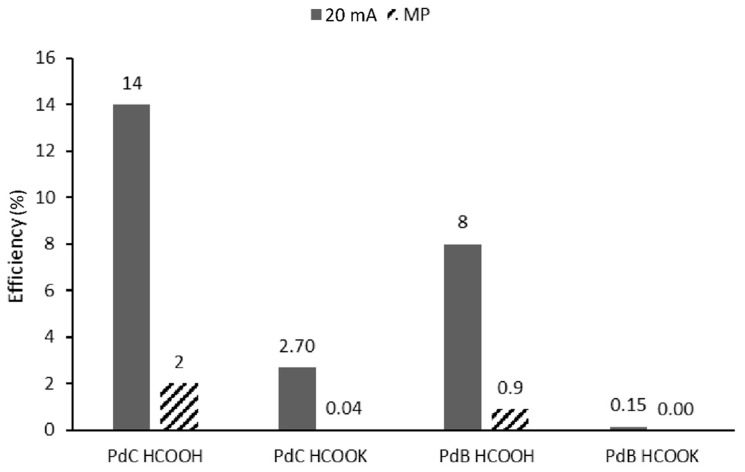
Constant current discharging efficiency at 20 mA and maximum power current (MP).

Moreover, power generation experiments were carried out to assess the performance of developed FCs in time. Performing the power generation experiments in constant current discharge, at maximum power peak, leads to unstable measurements due to fast potential losses [19]. Mikolajczuk et al., in his articles, proved the hypothesis that in high current densities the reaction never reaches the steady state and the CO_2_ bubble production is very fast, which leads to blocking of access of fuel to catalyst and deactivates the catalyst. In order to verify this assumption, in addition to the measurements at maximum power current, we performed experiments at 20 mA and indeed we observed higher efficiency in all studied cases (Figure 7).

Figure 7 reports the values obtained for all studied fuel cells’ configurations in power generation studies. As expected, based on polarization curves (Figure 5 and Figure 6), PdB has an overall lower efficiency of constant current discharge compared to PdC. On the other hand, the salt had high power density and current density but the energy production efficiency in constant current discharge was much lower. The FC efficiency is limited by catalyst activity, fuel dissociation state and by the solute conductivity. Above we showed that the dissociation of the salt is higher than acid, thus these differences can be assigned to the conductivity of cations. Indeed, limiting ion conductivity at 298.15 K of K^+^ is 73.50 S·cm^2^/mol, which is much lower than the one of H^+^ which is 349.85 S·cm^2^/mol [20]. Thus, even though the salt can be dissociated more easily, the overall power generation in time will be lower. Boncina et al. [20] observed that the molar conductivity of the salt does not change with its concentration, while for acid it exponentially decreases with the increase of molarity. This means that lower energy generation of salt could be compensated for with higher concentration.

Moreover, Nafion is a proton exchange membrane; therefore protons would pass through it easily. Yet, potassium ions might contribute to the membrane clogging, ultimately poisoning it. These assumptions are supported by studies reported by Bogdanowicz et al. [21]. In their article, among others, the authors studied the selectivity of cations’ permeability through Nafion 117 membranes and reported a higher transport resistance of K^+^ over H^+^, but it did not block the entrance.

## 4. Conclusions

MEA preparation with a home-made manual pressing system without heating with the Nafion glue application contributed to decreases of additional energy consumption. XRD analysis showed that palladium black and palladium on carbon are (111) face centered cubic structures, which makes them appropriate for formate/formic acid oxidation though HCOO* adsorption. PdB revealed low performance in polarization curves and exhibited a lower energy generation efficiency compared to PdC. Unsupported palladium agglomerated during the MEA preparation which caused decreases in mass transfer. Carbon supported Pd catalysts demonstrate good activity providing high contact area along with the potential for a more efficient Pd metal utilization with lower metal loadings.

Simple characterization in terms of the polarization curve does give us information about the capability of the system towards power generation. In this case, HCOOK gave better results due to higher dissociation constant over HCOOH. However, in the long term constant current discharge we observed a faster FC efficiency decrease when using potassium formate compared to formic acid. Based on the literature research it can be attributed to two possibilities, i) lower conductivity of potassium ion and ii) membrane clogging caused by K+. Hence, we proved the potential use of formate salt in passive, air breathing fuel cells, through HCOO* oxidation.

## Figures and Tables

**Table 1:** Fuel cell parameters.

Parameter	Value
Fuel cell nature	Passive
Anode	PdC or PdB, 1 mg/cm^2^
PEM	Nafion 117
Cathode	PtB, 4 mg/cm^2^
Catalyst effective area	9 cm^2^
Fuel	potassium formate or formic acid 1 M, 7.2 mL
Oxygen source	ambient air
Conditions	25 °C, 1013 hPa

**Table 2:** Theoretical reaction potential and measured open circuit potentials of the studied cells.

Theoretical Potential (V)	PdC OCP (V)		PdB OCP (V)	
	HCOOH 1 M	HCOOK 1 M	HCOOH 1 M	HCOOK 1 M
1.45	0.85	0.92	0.80	0.85

**Table 3:** Formate/formic acid fuel cells’ maximum power peak data.

Catalyst	PdC		PdB	
**Fuel (7.2 mL, 1 M)**	HCOOH	HCOOK	HCOOH	HCOOK
**Power max (mW/mg_Pd_)**	16.99	21.01	4.17	4.04
**Current at PM (mA/mg_Pd_)**	36.87	43.01	11.15	7.43

**Table 4:** Comparison of obtained results with literature findings.

Cathode	Anode (mg/cm^2^)	Membrane	Max. Power Density (mW/cm^2^)	T (°C)	Fuel and C (M)	O_2_ Source	Ref.
Fe–Co	Nano-Pd/C, 4	Commercial anion exchange	258	60	4 M HCOOK / 4 M KOH	Pure O_2_	[9]
Fe–Co	PdC, 2	Quaternized polysulfone	130	80	5 M HCOOK	Pure O_2_	[15]
PtB	PdB, 4	Polymer anion exchange	105	60	1 M HCOOK	Air (21%)	[16]
PtC	PdC, 4	Cation-exchange membrane	300	25	1 M HCOONa/3 M NaOH	H_2_O_2_	[17]
PtC	PdC, 2	Nafion membrane	103	45	3 M HCOOH	Pure O_2_	[18]
PtC	PdC, 1	Nafion membrane	5.6	25	1 M HCOOK	Air (21%)	This work

## Supplementary Materials

The following are available online at https://www.mdpi.com/2077-0375/10/11/355/s1, Table S1: List of abbreviations, Figure S1: Nafion membrane cleaning procedure, Figure S2: Home-made system for MEA fabrication, Figure S3: Catalytic ink.
