# Supplementary material for: MEA Preparation for Direct Formate/Formic Acid Fuel Cell—Comparison of Palladium Black and Palladium Supported on Activated Carbon Performance on Power Generation in Passive Fuel Cell
Source: Membranes (Basel). 2020 Nov 19;10(11):355. doi: 10.3390/membranes10110355 (PMC7699197; doi:10.3390/membranes10110355)
Supplement: Supplementary file 1 [file membranes-10-00355-s001.pdf]

Article

MEA preparation for direct formate/formic acid fuel cell – comparison of palladium black and palladium supported on activated carbon performance on power generation in passive fuel cell

Adrianna Nogalska [1*], Andreu Bonet Navarro [1, 2] and Ricard Garcia-Valls [1,2]

[1] Eurecat, Centre Tecnològic de Catalunya, C/Marcel·lí Domingo, Tarragona, 43007, Spain; adrianna.nogalska@eurecat.org (A.N.), andreu.bonet@eurecat.org (A.B.), ricard.garcia@eurecat.org (R.G.)

[2] Department of Chemical Engineering, Universitat Rovira I Virgili, Av. Països Catalans, 26, 43007, Tarragona Spain; andreu.bonet@estudiants.urv.cat (A.B.), ricard.garcia@urv.cat (R.G.),

* Correspondence: adrianna.nogalska@eurecat.org; Tel.: +34-977-297-089

Received: date; Accepted: date; Published: date

Table S1. List of abbreviations

Abbreviation	Full name
MEA	membrane electrode assembly
PEM	proton exchange membrane
CCM	catalyst coated membrane
GDE	gas diffusion electrode
GDL	gas diffusion layer
PdB	palladium black
PdC	palladium on activated carbon
MP	maximum power
FC	fuel cell
PMMA	polymethyl methacrylate
OCP	open circuit potential
HCOOH	Formic acid
HCOOK	Potassium formate,
DFAFC	Direct Formic Acid Fuel Cell
XRD	X-ray diffracion

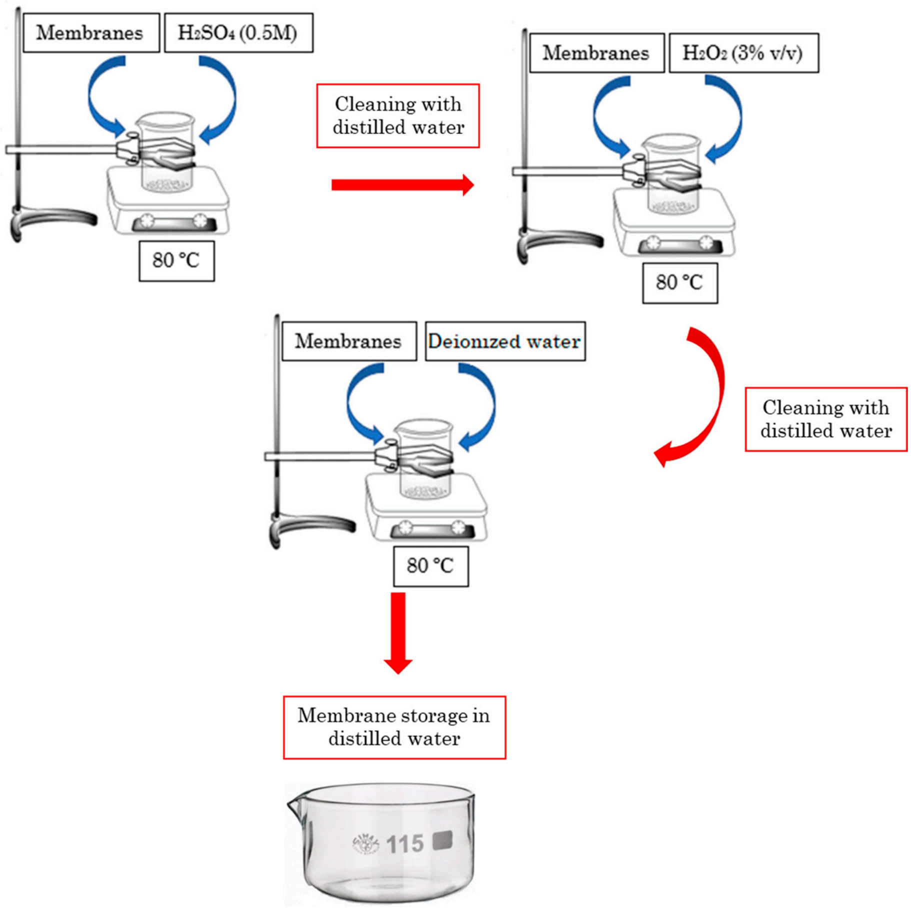

Figure S1. Nafion membrane cleaning procedure:

1.-Cut the membranes (using scissors) into pieces with desired size, but small enough to fit into the bottom of a 1l beaker.
2.-Immerge the membranes into 1l beaker with H_2SO_4 preheated up to 80 degrees with bath oil and leave them during 1h with stirring.
3.-Clean the membranes with distilled water immerging and shaking them into a big crystallizer before continuing with next step.
4.- Immerge the membranes into 1l beaker with H_2O_2 preheated up to 80 degrees with bath oil and leave them during 1h with stirring.
5.-Clean the membranes with distilled water immerging and shaking them into a big crystallizer before continuing with next step.
6.- Immerge the membranes into 1l beaker with distilled water preheated up to 80 degrees with bath oil and leave them during 1h with stirring.
7.-Store the membranes into a big crystallizer with distilled water (the membranes must be wet) and cover it with watch glass.

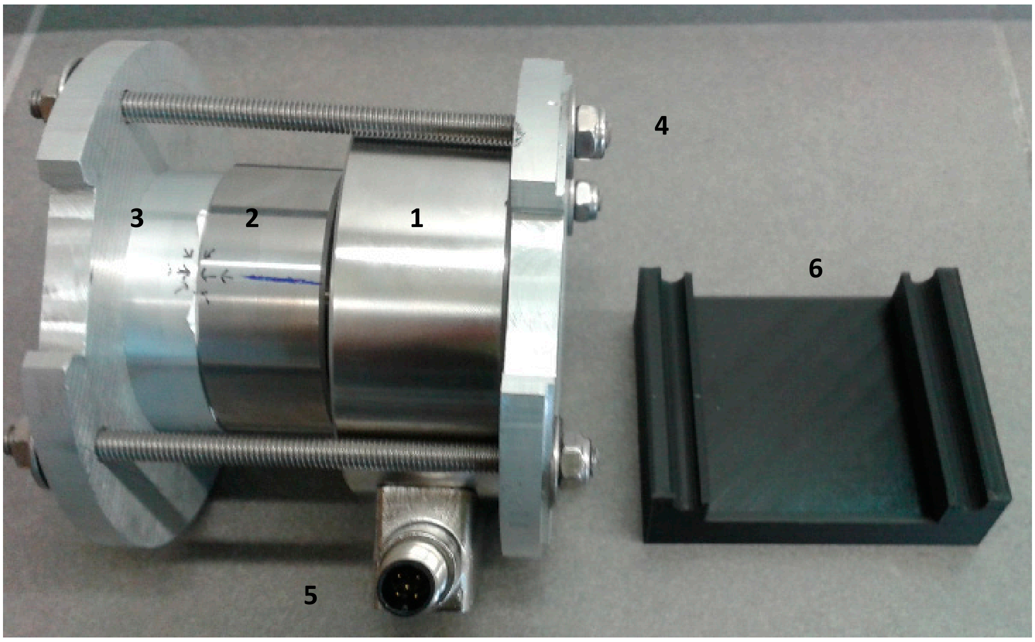

Figure S2. Home-made system for MEA fabrication. Devise an adjustment system is based on two plates of high hardness material, held by threaded rods in through holes at four opposite points (4). Inside the two plates is the load cell (1) with the flat-surface lug (2), and an additional part (3) of the same dimensions as the lug. The MEA placed between the additional piece and the stud is compressed by adjusting the screws always diagonally and measuring the value of the force exerted though the compression SENSING, S.L device connected in port (5). Once the required value is reached, the cell display can be disconnected.

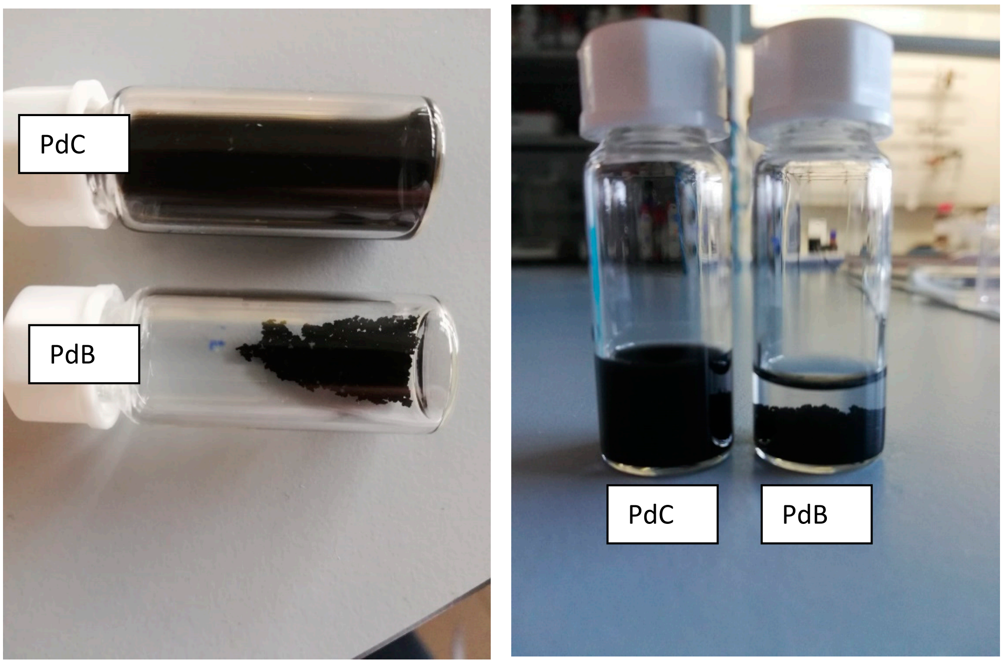

Figure S3. Catalytic ink. Comparison of homogeneous dispersion of catalyst in ink between PdC and PdB. The suspensions were sonicated in exaggerated time (2 h) to be able to appreciate the aggregation of particles of unsupported catalyst with naked eye.
